# Effect of Gadolinium Chloride on Liver Regeneration Following Thioacetamide-Induced Necrosis in Rats

**DOI:** 10.3390/ijms11114426

**Published:** 2010-11-04

**Authors:** Mirandeli Bautista, David Andres, María Cascales, José A. Morales-González, María Isabel Sánchez-Reus

**Affiliations:** 1 Área Académica de Farmacia, Instituto de Ciencias de la Salud, Universidad Autónoma del Estado de Hidalgo, Ex-Hacienda de la Concepción, Tilcuautla, 42080 Pachuca de Soto, Hgo, Mexico; E-Mail: jmorales101@yahoo.com.mx (J.A.M.-G.); 2 Instituto de Bioquímica (CSIC–UCM), Facultad de Farmacia, Ciudad Universitaria, Plaza de Ramón y Cajal S/N, 28040 Madrid, Spain; E-Mail: mireus@farm.ucm.es (M.I.S.-R.)

**Keywords:** gadolinium chloride, kupffer cells, thioacetamide hepatotoxicity, cell cycle

## Abstract

Gadolinium chloride (GD) attenuates drug-induced hepatotoxicity by selectively inactivating Kupffer cells. The effect of GD was studied in reference to postnecrotic liver regeneration induced in rats by thioacetamide (TA). Rats, intravenously pretreated with a single dose of GD (0.1 mmol/Kg), were intraperitoneally injected with TA (6.6 mmol/Kg). Hepatocytes were isolated from rats at 0, 12, 24, 48, 72 and 96 h following TA intoxication, and samples of blood and liver were obtained. Parameters related to liver damage were determined in blood. In order to evaluate the mechanisms involved in the post-necrotic regenerative state, the time course of DNA distribution and ploidy were assayed in isolated hepatocytes. The levels of circulating cytokine TNFα was assayed in serum samples. TNFα was also determined by RT-PCR in liver extracts. The results showed that GD significantly reduced the extent of necrosis. The effect of GD induced noticeable changes in the post-necrotic regeneration, causing an increased percentage of hepatocytes in S phase of the cell cycle. Hepatocytes increased their proliferation as a result of these changes. TNFα expression and serum level were diminished in rats pretreated with GD. Thus, GD pre-treatment reduced TA-induced liver injury and accelerated postnecrotic liver regeneration. No evidence of TNFα implication in this enhancement of hepatocyte proliferation and liver regeneration was found. These results demonstrate that Kupffer cells are involved in TA-induced liver damage, as well as and also in the postnecrotic proliferative liver states.

## 1. Introduction

Gadolinium chloride (GD) is a selective Kupffer cell toxicant that completely eliminates large Kupffer cells from the liver and has been extensively used in mechanistic studies of hepatotoxic processes [1]. Kupffer cells, as the macrophages residing in the sinusoids of the liver, are the first macrophage population to come into contact with drugs. These cells are anchored to the endothelium in the lumen of the sinusoids [2]. Kupffer cells exhibit intraacinar heterogeneity, since those located in the periportal area are larger and exhibit higher phagocytic activity compared with those located in the perivenous area [3]. It is well known that the function of these cells (release of cytokines and proteases, superoxide anion production, *etc*.) plays an important role in the pathogenesis induced by hepatotoxic compounds [4]. GD, most likely, is protective because it prevents the release of inflammatory cytokines and toxic oxygen radicals produced by activated Kupffer cells [5,6].

Thioacetamide (TA) is a potent hepatotoxic agent which, when administered at doses of 500 mg/Kg to rats, initiates a severe hepatocellular perivenous necrosis [7,8]. The selective destruction of perivenous hepatocytes and the proliferative state of liver cells that immediately follows have been used as an experimental model by which to study the hepatic response against the aggressive attack of a hepatotoxic drug. Thus, this response presents a double aspect: the hepatocellular necrosis and the post-necrotic hepatocellular regeneration linked to the restoration of liver function [9,10].

Kupffer cells are also the major source of mitogens, such us tumor necrosis factor α (TNFα), in the liver [11,12]. TNFα is a multifunctional cytokine [13] that acts as a mediator of the acute phase response in the liver and is a cytotoxic agent in many types of hepatic injury. Some authors have suggested that TNFα may be necessary for hepatocyte proliferation [13]. The observation that TNFα is required for liver regeneration is surprising because TNFα is a proinflammatory cytokine and a mediator of the acute phase response. The proliferative and anti-apoptotic effect of this cytokine seems to take place only under special conditions, such as those existing after partial hepatectomy. Although TNFα appears to be beneficial and required for liver regeneration after partial hepatectomy, the necessity of this factor has not been as clearly established after liver injury (a more common regenerative stimulus.) In fact, a number of studies have suggested that TNFα increases liver injury after toxic damage [14]. Moreover, [15] demonstrated that the absence of TNFα does not impair liver regeneration.

The role of GD in TNFα expression by Kupffer cells has been widely debated. The depletion of Kupffer cells, the major source of TNFα production in the liver, should initiate a decrease in serum TNFα and mRNA TNFα level in the liver, a fact that has been described and corroborated by several authors [6,16–19]. However, other authors have reported opposite data [1,20,21] after partial hepatectomy in rats pretreated with GD. Moreover, depletion of Kupffer cells with GD seems to increase hepatocyte proliferation and liver regeneration following partial hepatectomy [20,22]; however, the mechanism responsible remains unknown.

As it is generally accepted that the Kupffer cell function is involved in the severity of liver damage induced by drugs, and that GD induces a selective blockade of Kupffer cell function when administered intravenously, the purpose of the present study is to elucidate the role of Kupffer cells in regeneration after liver injury, blocking specifically Kupffer cell function by GD. The effect of GD was assayed on an experimental model of liver injury induced by a single necrogenic dose of TA which results in necrosis in the perivenous acinar area. Groups of rats were pre-treated or not intravenously with GD 24 h before TA. The proliferative post-necrotic response was assayed by evaluating the ploidy and DNA distribution in the cell cycle phases in isolated hepatocytes by flow cytometry.

## 2. Materials and Methods

### 2.1. Reagents

Enzymes were obtained from Boehringer Mannheim (Mannheim, Germany). Substrates and coenzymes were from Sigma (St Louis, MO, USA). Standard analytical grade laboratory reagents were obtained from Merck (Darmstadt, Germany). Antibodies for Western-blot analysis were obtained from Santa Cruz Biotechnology.

### 2.2. Animals and Treatment

Two months old male Wistar rats (200–220 g) were obtained from PANLAB (Barcelona), and acclimated to our animal room for two weeks during which time rats were supplied with food (SanderSA) and water *ad libitum,* exposed to a 12 h light-dark cycle and given intraperitoneally a single necrogenic dose of thioacetamide (6.6 nmol/Kg body weight) freshly dissolved in 0.9% NaCl. The dose of thioacetamide was chosen as the highest dose with survival above 90% [23,24]. GD pre-treatment was performed 24 h before thioacetamide. GD was dissolved in 0.9% NaCl and injected in a tail vein (0.1 mmol/Kg body weight). Untreated animals received 0.5 mL of 0.9% NaCl. Hepatocytes were isolated from rats by the classic perfusion method [25] at 0, 12, 24, 48, 72 and 96 h following thioacetamide. Samples of blood and liver were also obtained. Experiments were performed on two different groups: rats treated with a single dose of thioacetamide (TA) and rats pre-treated with GD and treated with a single dose of thioacetamide (GD + TA). Each experiment was performed in duplicate from four different animals and followed the international criteria for the use and care of experimental animals outlined in T*he Guiding Principles in the use of Animals in Toxicology* adopted by the Society of Toxicology in 1989.

### 2.3. Processing of the Samples

In order to clarify the sequential changes during the different stages of liver injury and the post-necrotic regenerative response, samples were obtained from controls at 12, 24, 48, 72, and 96 h of TA intoxication from both GD pre-treated or non pre-treated animals. Rats were cervically dislocated and samples of liver were obtained and processed as previously described [26]. Blood was collected from hearts and kept at 4 °C for 24 h, centrifuged at 3000 rpm for 15 min, and serum was obtained as the supernatant. Hepatocytes were isolated by the collagenase perfusion technique as previously described [25]. The viability of isolated hepatocytes (>90%) was assessed by trypan blue exclusion as previously described [27].

### 2.4. Determination of Parameters of Injury and TNFα in Serum

As a marker of necrosis, serum aspartate aminotransferase (AST, EC 2.6.2.1) activity was spectrophotometrically measured at 340 nm in the presence of a-ketoglutarate, aspartate [28]. TNFα was assayed in serum using the Biotrak ™ [(r)TNFα] ELISA system (Amersham Pharmacia Biotech).

### 2.5. RT-PCR Analysis of TNFα

For RT-PCR, total RNA (1 μg) was subjected to random primed first-strand cDNA synthesis in 40 μL reactions composed of 50 mM Tris-HCl, 75 mM KCl, 3 mM MgCl2, 10 mM DTT, 1 mM dNTPs (each), 50 ng of random hexamer, 0.5 IU/μL Mo-Mu-LV reverse transcriptase (Super-Script Pre-Amplification System; Gibco-BRL, Life Technologies). The reactions were incubated for 60 min at 42 °C and terminated at 65 °C for 15 min. The first-strand cDNAs were subsequently amplified by PCR; β-actin cDNA was used as an internal control. The sequences of the primers were as follows: TNFα sense: 5’-TGG CCC AGA CCC TCA CAC TC-3’; TNFα antisense: 5’-CTC CTG GTA TGA AAT GGC AAA TC-3’; β-actin sense: 5’-TAC AAC CTC CTT GCA GCT CC-3’; β-actin antisense: 5’-GGA TCT TCA TGA GGT AGT CAG TC-3’. The PCR reaction mixture contained PCR buffer (20 mM Tris-HCl [pH 8.4], 50 mM KCl), 1.5 mM MgCl2, 100 μM dNTPs (each), 0.4 μM primers and 0.0025 U/μLTaq polymerase in a final volume of 50 μL. The number of PCR cycles was adjusted to avoid saturation of the amplification system (94 °C for 1 min, at 59 °C for 1 min and 72 °C for 1 min (35 cycles) for TNFα and 94 °C for 30 s, 58 °C for 45 s and 72 °C for 30 s (24 cycles) for β-actin) with a final elongation at 72 °C for 10 min. Amplification products were visualized on 1.8% agarose gels containing ethidium bromide (1 μg/mL): TNFα product, 281 bp; β-actin product 630 bp. A 100 bp DNA ladder was used as marker. The products were quantified by laser densitometer.

### 2.6. Flow Cytometry Analysis of DNA Content

10^6^ isolated viable hepatocytes were stained with propidium iodide following the multistep procedure of [29]. The emitted fluorescence of the DNA-propidium iodide complex was assayed in a FACScan flow cytometer (Becton-Dickinson) in the FL2-A channel. A double discriminator module was used to distinguish between signals coming from a single nucleus and those products of nuclear aggregation. Data analysis was carried out by means of evaluation of single inputs (10^4^ nuclei/assay) and was expressed as percent of DNA distribution in the cell cycle phases G_0_/G_1_ (2C), S_1_, G_2_ + M (4C), S_2_, (G_2_ + M)_2_ (8C), and hypodiploid peak (<2C).

### 2.7. Statistical Analysis

The results were calculated as the means ± SD of four experimental observations in duplicate (four animals). Differences between groups were analyzed by an ANOVA following Snedecor F (α = 0.05). Student’s t test was performed for statistical evaluation as follows: (a) all values against their control; (b) differences between two groups GD + TA *versus* TA.

## 3. Results

### 3.1. Effect of GD on Parameters of Liver Necrosis

**Figure 1.**
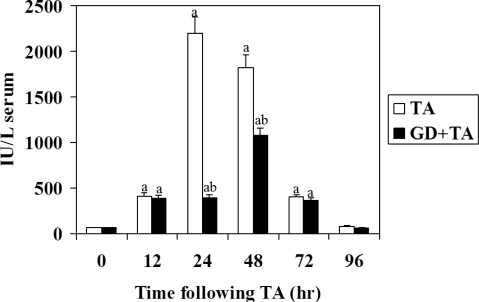
Effect of GD pre-treatment on aspartate aminotransferase (AST) activity in serum of rats intoxicated with one sublethal dose of thioacetamide (TA). Samples were obtained at 0, 12, 24, 48, 72 and 96 h following TA. The results, expressed as nmol per min per ml of serum, are the mean ± SD of four determinations in duplicate from four rats. Differences against the respective control are expressed as (a) and differences due to GD are expressed as (b) *p* < 0.05.

Liver damage induced by xenobiotics is characterized by the release of hepatic enzymes in serum due to necrosis of hepatocytes. AST is randomly distributed in the hepatic acinus, and is the enzyme activity used as a marker of necrosis. An increase in AST was detectable at 12 h after TA administration and reached maximum at 24 h (Figure 1). The extent of necrosis induced by TA was detected by a peak of 30-times the basal values, for AST activity. When rats were pretreated with GD the 24 h peak was reduced to 15%. However, at 48 hours of intoxication, the difference due to GD was 56% for this enzyme activity, indicating that GD delays TA-induced liver injury, since the maximum necrosis appeared at 48 h of intoxication. No effects were detected on serum activities when GD was administered without TA (data not shown).

### 3.2. Effect of GD Pretreatment on the Time Course of Genomic DNA Ploidy and Distribution in Hepatocytes Isolated from TA-Treated Rats

**Figure 2.**
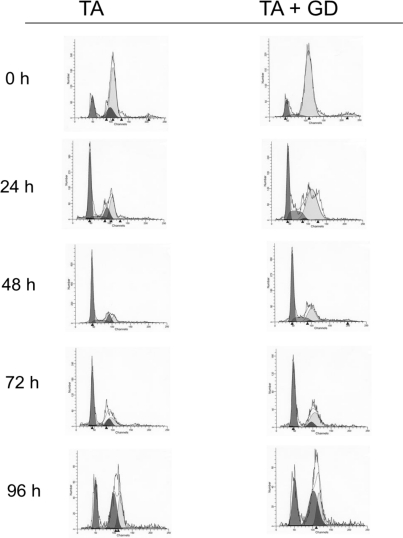
Representative histograms of the DNA content determined on the basis of fluorescence emission at 623 nm by the DNA-propidium iodide complex. These histograms are expressed as the relative number of cells (vertical axis) plotted against the fluorescence (horizontal axis) that represents the DNA content. The quantitative values of Figure 2 appear in Table 1 which also shows the percentages of cell cycle populations related to ploidy and DNA content.

Figure 2 shows representative histograms of the DNA content determined on the basis of fluorescence emission at 623 nm by the DNA-propidium iodide complex. These histograms are expressed as the relative number of cells (vertical axis) plotted against the fluorescence (horizontal axis) that represents the DNA content. The quantitative values of Figure 2 appear in Table 1, which also shows the percentages of cell cycle populations related to ploidy and DNA content.

Following TA, liver cells exhibited marked variations in the pattern of DNA distribution which can be summarized as a sharp decrease at 48 h in tetraploid population parallel to an increase in diploid population, followed by restoration to close to normal values at 96 h. The S_1_ population also increased from 24 h reaching a maximum at 48 h. When rats were pretreated with GD, the variations in the pattern of DNA distribution was very similar to that observed in TA group, however, with an important difference: the highest increase in S_1_ population was reached at 24 h (17.17% *versus* 10.01%) instead of at 48 h, so the proliferative state in the hepatocytes was reached 24 h before that obtained in the rats treated with the single dose of TA.

### 3.3. Effect of GD Pretreatment on Serum TNFα Level and TNFα Expression in Rat Liver Following Intoxication with TA

**Figure 3.**
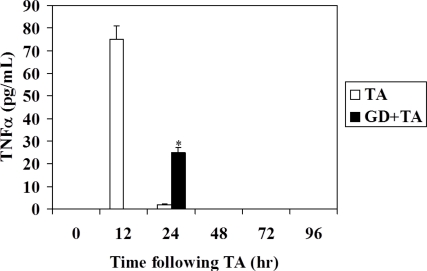
Effect of GD pre-treatment on the levels of TNFα in serum of rats intoxicated with thioacetamide. The results, expressed as pg/mL of serum, are the mean ± SD of four determinations in duplicate from four rats. Differences between GD pre-treated or non-pretreated rats are expressed as *p < 0.05.

TNFα is a multifunctional cytokine that acts in the liver as a mediator of the acute phase response and is a cytotoxic agent in many types of hepatic injury. In serum of rats intoxicated with TA, with or without GD pretreatment, TNFα was assayed. Figure 3 shows the levels of serum TNFα. Following TA, the level of this cytokine increased markedly in serum at 12 h after intoxication, and when GD was preadministrated, TNFα was significantly lowered and appeared at 24 h following TA.

**Figure 4.**
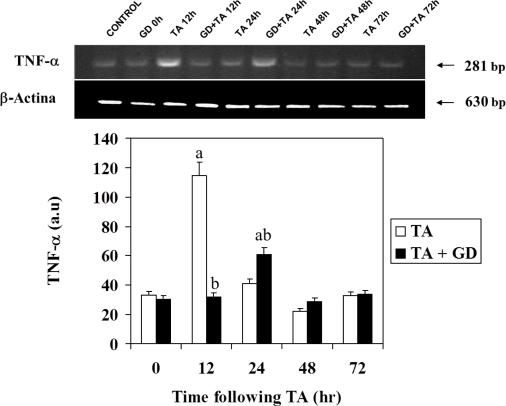
Effect of GD pre-treatment on the levels of TNFα assayed by RT-PCR analysis in liver homogenates of rats intoxicated with a sublethal dose of thioacetamide. Samples were obtained at 0, 12, 24, 48 and 72 h. The results are the mean ± SD of four determinations from four rats (in arbitrary units). Differences against the respective control are expressed as (a) and differences due to GD are expressed as (b), *p* < 0.05.

Figure 4 shows the levels of TNFα mRNA assayed by RT-PCR. As shown in serum TNFα, the levels of mRNA follow the same pattern, which corroborates the results obtained by ELISA in serum.

## 4. Discussion

TA-induced liver injury is a well established area of considerable pharmacological interest, since reactive oxygen species and free radicals, generated in the microsomal drug oxidation, participate in the mechanisms of cell death [26,30,31]. In the present study, TA-induced hepatotoxicity was used to investigate the effect of a single dose of GD, previously administered, on the multistep events involved in liver regeneration. Previous reports described that when TA was intraperitoneally administered to rats, necrosis developed and peaked at 24 h of intoxication, and that a synchronous proliferative response was immediately initiated reaching a peak of DNA synthesis at 48 h [26]. The postnecrotic proliferative response, after experimental liver cell death, constitutes an interesting area in which to study the factors involved in the regulation of hepatocyte proliferation. The results obtained in the present paper provide evidence that GD, when administered intravenously prior to TA, significantly enhances liver regeneration.

The study of enzyme systems in serum obtained at different times after administration of thioacetamide to rats pretreated or not with GD, shows that the hepatocellular necrosis induced by thioacetamide changes significantly by the effect of pretreatment with gadolinium. One of these enzymatic systems in serum, the aspartate aminotransferase, marker of hepatocellular necrosis, showed a maximum at 24 h after administration of hepatotoxic (30-times the control). However, pretreatment with GD not only delayed the peak of the injury, but had significantly lower values. The variations observed by effect of pretreatment with GD were significant in the enzyme assayed at 24 and 48 hours of intoxication. It should be noted that the maximum necrosis in the liver pretreated with GD was detected at 48 h; 24 h after the maximum peak of damage produced by thioacetamide. The activity of this enzyme decreased showing, at 96 hours of treatment, values close to control.

Otherwise, it is well known that flow cytometry offers an important tool to evaluate the genomic DNA cell populations whose proliferation is impaired, as in tumor tissues or in hepatocytes which have suffered necrogenic damage. By this means, one can detect different populations of hepatocytes according to their ploidy and distribution of cell cycle phases, remaining hepatocytes, hepatocytes dedifferentiated proliferating and newly divided hepatocytes. In the model of necrosis-regeneration of this work, the changes we have observed provide evidence on the following phenomena:

- That pretreatment with GD increases the cellular dedifferentiation induced by thioacetamide,
- That the analogy with the fetal pattern in hepatocellular injury is more severe in the group pretreated with GD.(A) The liver of mammals contains polyploid hepatocytes, whose number depends on the species and age of the animal [32]. Fetal rat hepatocytes are mostly diploid with an allocation of 85.3% of cells involved in this phase (diploid), 7.3% in DNA synthesis phase and 7.4% polyploid (tetraploid + octoploid) [9]. Fetal liver cells of animals have a greater number of cells in S (S_1_ and S_2_) and diploid phases and fewer diploid and polyploid (tetraploid + octoploid). In adult animals, the polyploidization increases and the synthesis is reduced to values of approximately 1%.

So far, there is no clear explanations regarding the physiological significance of ploidy increase by effect of age, but it is suspected to be a reflection of increasing degrees of cell differentiation and the requirement of hepatocytes with large amounts of gene product for its multifunctional metabolic role. Polyploid cells may be mononuclear and binucleated; the mononuclear being the most differentiated in development.

(B) The necrosis-regeneration process is characterized by a change in the distribution of different populations of hepatocytes and that while normal hepatocytes have stability in their DNA content and chromosome organization, proliferative state of hepatocytes (regenerating) are involved in the replication process through the cell cycle including DNA synthesis programmed to duplicate the genomic material of the cell before its division. Regenerating cells, therefore, have a higher DNA content than quiescent cells (resting phase) and this content can vary in each cycle according to the progression of DNA synthesis.

The process of liver regeneration has been, for several decades, the subject of intense studies by which it is known that the liver responds to an attack that involves an hepatocellular necrosis, inducing liver cell division on remaining hepatocytes. Thus, the damaged liver can recover as much mass as the initial cell number in a short period of time. For many years it was believed that the loss of hepatocellular mass was the agent that triggered the remaining cells division, but several authors [33–35] have observed that there may be hepatocellular regeneration without any loss in mass or number of liver cells, indicating that the precipitating agent has to be an indicator of liver function and only when this feature is satisfied there is a cessation of cell proliferation and ends regeneration.

Although hepatocytes are highly differentiated cells that are in quiescent phase, they have the ability to divide to replace cells that have been damaged. Under normal conditions the proliferative capacity of the liver is very low and the hepatocytes are in G_0_/G_1_ phase of the cell cycle, however, the liver is one of the body's most transcriptionally active tissues. The proliferation of the liver parenchyma begins in the periportal areas and then moves toward perivenous areas. Thus, within two to three weeks, the remaining liver segment retrieves the number of cells and the original weight of the liver. In the course of this compensatory hyperplasia, 90% of hepatocytes are divided between 24 and 48 hours after hepatectomy [34,35]. The regional selectivity of necrosis must necessarily influence the regeneration process, in fact, it has long been known that the periportal region is the most qualified in the proliferative process [36], and, therefore, in the case of thioacetamide-induced necrosis, proliferation occurs through a dedifferentiation of adult hepatocytes that acquire the characteristics of fetal hepatocytes [9].

In this paper, we used a classic necrosis-regeneration model, as is induced by thioacetamide administration, widely studied and known by our group, to which we added a variation: pretreatment with GD, an inhibitor of Kupffer cells, allowing us to establish the degree of contribution of these cells in the complex process of injury and liver regeneration. To study the regenerative process, the flow cytometry technique was used in order to determine the distribution of genomic DNA in different cell cycle phases and ploidy; in control hepatocytes population, hepatocytes in which division is induced by thioacetamide (TA) and in hepatocytes pretreated with GD and subsequently treated with thiacetamide (GD + TA).

The results obtained by this technique, show that in the group of rats treated with thioacetamide the population of hepatocytes with diploid chromosome at the time of necrosis (24 h) increased, peaking at 48 hours (50.86% *versus* 13.0% of control), to decrease at 72 and 96 hours. The population of tetraploid hepatocytes evolves as opposed to that the diploid population, so that it reaches a minimum at 48 hours (23.66% *versus* 78.35% of control) in order to increase population at 72 and 96 hours. These data are in total agreement with previous results from our group [9,10], as well as with data on the DNA synthesis phase of S_1_, resulting in significant increases from 24 h and reaching the maximum at 48 h for later return to values near control. Interestingly, the increase in DNA replication coincides with a sharp decline in the polyploid population, which may be indicative of an even more accelerated mitotic DNA synthesis. Despite the decline in this population may also be due to necrosis mainly affects the cells located in G_2_/M cell cycle.

On the other hand, an increase in liver cells of diploid chromosome is accompanied by a decrease in tetraploid and octoploid hepatocytes. The increase in the diploid fraction in mature hepatocytes indicates a regression to less differentiated states typical of developing or neoplastic cells, in which the ploidy profile of characteristics acquired fetal hepatocytes [8].

Moreover, the data belonging to the group of rats pretreated with GD, to suppress the activity of Kupffer cells, show a similar profile to that shown in rats treated with thioacetamide alone. However, there is a higher significant increase in the population of cells that are undergoing DNA synthesis at 24 h (17.17% *versus* 10.01% in rats treated with thioacetamide), which coincides with the maximum value, so it is after 24 h when the population in S_1_ phase starts to decline, whereas in the case of rats treated with thioacetamide it is at 48 hours when this phase starts to descent. This apparent advancement in the initiation of cell proliferation in rats pretreated with GD seems to agree with results reported by other authors in models of liver regeneration induced by partial hepatectomy [1,20]. However, the mechanisms responsible for these changes in liver regeneration remain unknown. Several theories have been proposed to explain this fact, including that proposed by Rai *et al*. [21] which exposed that GD would lead to an altered balance between ***pro-mitogenic*** and ***anti-mitogenic*** cytokines in the liver. In this section there is a point of controversy about the role GD can play on the expression of tumor necrosis factor alpha (TNFα).

TNFα is a multifunctional cytokine [37] that signals through two distinct receptors TNFR-1 and TNFR-2. In the liver, TNFα mediates acute phase response and is a cytotoxic agent in various types of liver damage [13]. However, it has been shown [38], that the administration of TNFα antibodies inhibits liver regeneration after partial hepatectomy in rats, suggesting that TNFα may be necessary in the proliferation of hepatocytes. The observation that TNFα is necessary in liver regeneration is surprising, since it is a proinflammatory cytokine and a mediator of acute phase response [39]. It appears that the proliferative effect of TNFα occurs only under special conditions such as those that occur in the event of partial hepatectomy [13]. When high doses of TNFα injected in rats induces cell replication in the liver, however, low doses of the same cytokine have no proliferative effect [13]. These considerations lead to the hypothesis that TNFα does not have a full mitogenic capacity, but acts as an agent and starts preparing hepatocytes for cell replication and starts making these cells more responsive to the effect of growth factors [13]. Kupffer cells are the main source of TNFα production in the liver [12,40,41], so it would assume that the inhibition of Kupffer cell function by GD would result in a decrease in the expression levels of TNFα. Several authors have published results that corroborate this hypothesis *in vivo* [16–18,40], however, other authors, using different experimental models *in vivo*, as well as *in vitro*, have found completely opposite results, such as treatment with GD causes induction of TNFα expression and increased plasma levels [1,20,21]. According to these authors, the increased expression of TNFα was responsible for the advancement in the initiation and completion of cell proliferation detected in the experiments of this study in rats pretreated with the inhibitor of Kupffer cells.

The hypothesis proposed by other authors who assigned a crucial role in the advancement of TNFα and acceleration of cell proliferation, is ruled out in our experimental model, since the release of this cytokine decreases significantly by inhibiting the function of Kupffer cells, the main source of TNFα in the liver. It is also proposed that metallothionein may play an important role in the regeneration pattern observed in this study. However, the effects observed in the regeneration cannot be attributed exclusively to the changes seen in the proteins studied in this work, as the complexity of the process of liver regeneration, which involve numerous cytokines whose signaling pathways are not completely understood at present.

On the other hand, several authors [42,43] have assigned an important role in liver regeneration after partial hepatectomy or after necrosis induced by hepatotoxic agents to metallothionein; a finding that the maximum levels of metallothionein match with the peak of hepatocellular proliferation and concluding that the differences in the distribution of metallothionein after liver injury are directly reflected in the proliferation capacity of the liver. Previous studies have shown that indeed there is an increase of metallothionein levels during liver necrosis and regeneration induced by TA. Moreover, studies from our group [6] have shown that pretreatment with GD *per se* increases MT levels as seen in the control of GD. So that higher levels of metallothionein in rats treated with GD may explain the higher regeneration and acceleration in the growth observed in these rats after necrosis induced by thioacetamide. All this suggests that induction of metallothionein expression by GD may be part of the mechanisms by which this inhibitor of Kupffer cells accelerates liver regeneration.

In summary, the results show that thioacetamide hepatotoxicity enhances the activity of Kupffer cells, which is reflected in an increase in the degree of hepatocellular necrosis and oxidative stress and increase of the expression of stress proteins like metallothionein. It was also found that thioacetamide induces expression and release of TNFα, and myeloperoxidase activity in serum. These cytokines and myeloperoxidase activity are mainly produced by Kupffer cells and are involved in the programming of liver damage product of the inflammatory response. Blocking the function of Kupffer cells by GD apparently interrupted a step in the sequence of events leading to hepatotoxicity.

## 5. Conclusion

We conclude that gadolinium pre-treatment significantly enhances liver regeneration after thioacetamide-induced hepatotoxicity. The mechanism by which this attenuation is verified is both by direct inhibiting of Kupffer cell function and through inhibiting thioacetamide biotransformation, thus reducing the ability of this hepatotoxic compound to activate phagocytic cells. In our experimental conditions, the degree of this attenuation is close to 50%. The modulation of Kupffer cell function by GD may serve as a potential target for therapeutics and could be useful for preventing liver damage induced by drugs.

## Figures and Tables

**Table 1.** Quantitative analysis of DNA ploidy.

	**Hypodiploid (**<**2C)**	**Diploid (2C)**	**S_1_ Phase (2C** → **4C)**	**Tetraploid (4C)**	**S_2_ Phase (4C → 8C)**	**Octoploid (8N)**
**Control**	0.54	13.0	1.1	78.35	2.62	4.35
**Control Gd**	0.70	17.86	0.9	72.17	4.60	3.72
**TA 24**	2.80	42.76^a^	10.01^a^	40.2ª	6.72ª	0.30
**TA-Gd 24**	1.36	26.15^a^^b^	17.17^a^^b^	50.96^a^^b^	4.32	0.32
**TA 48**	1.66	50.86^a^	14.45^a^	23.66^a^	9.18^a^	0
**TA-Gd 48**	2.35	42.77^a^	14.92^a^	28.0^a^	10.25ª	1.70
**TA 72**	4.08ª	46.11^a^	6.89^a^	36.32ª	4.49ª	2.12
**TA-Gd 72**	2.86ab	47.98^a^	1.74^b^	40.61ª	6.26	0.53
**TA 96**	1.17	23.94^a^	4.98^a^	60.56	9.06ª	0.29
**TA-Gd 96**	1.15	28.56^a^	0	63.65	6.62	0.02

The values are expressed as the percentage of DNA in: hypodiploid population (<2C), G_0_/G_1_ diploid population (2C), S_1_ population (2C → 4C), G_2_ + M tetraploid population (4C), S_2_ population (4C → 8C) and (G_2_ + M)_2_ octoploid population. Data are reported as the mean ± SD of four different observations (four animals). Differences between groups were analyzed by an ANOVA following Snedecor F (α = 0.05). Student’s t test was performed for statistical evaluation as follows:

(a) all values against their control;

(b) differences between two groups GD + TA *vs.* TA.

## References

[1] Rose ML, Bradford BU, Germolec DR, Lin M, Tsukamoto H, Thurman RG (2001). Gadolinium chloride-induced hepatocytes proliferation is prevented by antibodies to Tumor Necrosis Factor α. Toxicol. Appl. Pharmacol.

[2] Laskin DL (1990). Nonparenchymal cells and hepatotoxicity. Semin. Liver Dis.

[3] Bautista AP, Skrepnic N, Niesman MR, Bagby GJ (1994). Elimination of macrophages by liposome-encapsulated dichloromethylene diphosphonate suppresses the endotoxin-induced priming of Kupffer cells. J. Leukoc. Biol.

[4] Ding H, Peng R, Reed E, Li QQ (2003). Effects of Kupffer cell inhibition on liver function and hepatocellular activity in mice. Int. J. Mol. Med.

[5] Zhong Z, Connor HD, Mason RP, Qu W, Lemasters JJ, Thurman RG (1995). Role of Kupffer cells in reperfusion injury in fat-loaded livers from ethanol-treated rats. J. Pharmacol. Exp. Ther.

[6] Andres D, Sanchez-Reus I, Bautista M, Cascales M (2003). Depletion of Kupffer cell function by gadolinium chloride attenuates thioacetamide-induced hepatotoxicity. Expression of metallothionein and HSP70. Biochem. Pharmacol.

[7] Landon EJ, Naukam RJ, Rama Sastry BV (1986). Effects of calcium channel blocking agents on calcium and centrilobular necrosis in the liver of rats treated with hepatotoxic agents. Biochem. Pharmacol.

[8] Cascales M, Martín-Sanz P, Alvarez A, Sanchez-Pérez M, Díez-Fernández C, Boscá L (1992). Isoenzymes of carbohydrate metabolism in primary cultures of hepatocytes from thioacetamide-induced rat liver necrosis: Responses to growth factors. Hepatology.

[9] Diez-Fernandez C, Boscá L, Fernandez-Simón L, Alvarez A, Cascales M (1993). Relationship between genomic DNA parameters of liver damage during necrosis and regeneration induced by thiacetamide. Hepatology.

[10] Sanz N, Diez-Fernández C, Andrés D, Cascales M (2002). Hepatotoxicity and aging: endogenous antioxidant systems in hepatocytes from 2-, 6-, 12-, 18- and 30-month-old rats following a necrogenic dose of thioacetamide. Biochim. Biophys. Acta.

[11] Decker K (1990). Biologically active products of stimulated liver macrophages (Kupffer cells). Eur. J. Biochem.

[12] Olynyk JK, Matuschak GM, Lechner AJ, Britton RS, Tredway TL, O'Neill R, Bacon BR (1994). Differential production of TNF by Kupffer cells after phagocytosis of *E. coli* and *C. albicans*. Am. J. Physiol.

[13] Fausto N (2000). Liver Regeneration. J. Hepatol.

[14] Taub R (2004). Liver regeneration: From myth to mechanism. Nat. Rev. Mol. Cell. Biol.

[15] Fujita J, Marino MW, Wada H, Jungbluth AA, Mackrell PJ, Rivadeneira DE, Stapleton PP, Daly JM (2001). Effect of TNF gene depletion on liver regeneration after partial hepatectomy in mice. Surgery.

[16] Iimuro Y, Yamamoto M, Kohno H, Itakura J, Fujii H, Matsumoto Y (1994). Blockade of liver macrophages by gadolinium chloride reduces lethality in endotoxemic rats. Analysis of mechanisms lethality in endotoxemia. J. Leukoc. Biol.

[17] Lazar G, Lazar G, Kaszaki J, Olah J, Kiss I, Husztik E (1994). Inhibition of anaphylactic shock by gadolinium chloride-induced Kupffer cell blockade. Agents Actions.

[18] Fujita S, Arii S, Monden K, Adachi Y, Funaki N, Higashitsuji H, Furutani M, Mise M, Ishiguro S, Kitao T (1995). Participation of hepatic macrophages and plasma factors in endotoxin-induced liver injury. J. Surg. Res.

[19] Kono Y, Fridovich I (1982). Superoxide radical inhibits catalase. J. Biol. Chem.

[20] Rai RM, Yang SQ, McClain C, Karp CL, Klein AS, Diehl AM (1996a). Kupffer cell depletion by gadolinium chloride enhances liver regeneration after partial hepatectomy in rats. Am. J. Physiol.

[21] Rai RM, Zhang JX, Clemens MG, Diehl AM (1996b). Gadolinium chloride alters the acinar distribution of phagocytosis and balance between pro- and anti-inflammatory cytokines. Shock.

[22] Rai RM, Loffreda S, Karp CL, Yang SQ, Lin HZ, Diehl AM (1997). Kupffer cell depletion abolishes induction of interleukin-10 and permits sustained overexpression of tumor necrosis factor alpha messenger RNA in the regenerating rat liver. Hepatology.

[23] Sanz N, Díez-Fernández C, Alvarez AM, Fernández-Simón L, Cascales M (2000). Age-related changes on parameters of experimentally-induced liver injury and regeneration. Toxicol. Appl. Pharmacol.

[24] Zaragoza A, Andrés D, Sarrión D, Cascales M (2000). Potentiation of thioacetamide hepatotoxicity by phenobarbital pretreatment in rats. Inducibility of FAD monooxygenase system and age effect. Chem. Biol. Interact.

[25] Seglen PO, Tyson CA, Frazier JM (1993). Isolation of hepatocytes by collagenase perfusion. Methods in Toxicology In vitro Biological Systems.

[26] Sanz N, Díez-Fernández C, Fernández-Simón L, Alvarez A, Cascales M (1998). Necrogenic and regenerative responses of liver of newly weaned rats. Biochem. Biophys. Acta.

[27] Diez-Fernandez C, Sanz N, Cascales M (1996). Changes in glucose-6-phosphate dehydrogenase and malic enzyme gene expression in acute hepatic injury induced by thioacetamide. Biochem. Pharmacol.

[28] Ramírez-Farías C, Madrigal-Santillán E, Gutiérrez-Salinas J, Rodríguez-Sánchez N, Martínez-Cruz M, Valle-Jones I, Gramlich-Martínez I, Hernández-Ceruelos A, Morales-González JA (2008). Protective effect of some vitamins against the toxic action of ethanol on liver regeneration induced by partial hepatectomy in rats. World J. Gastroenterol.

[29] Vindelov LL, Christensen IJ, Nissen NI (1983). A detergent trypsin method for the preparation of nuclei for flow cytometric. Cytometry.

[30] Cascales M, Martin-Sanz P, Craciunescu DC, Mayo I, Aguilar A, Robles-Chillida EM, Cascales C (1991). Alterations in hepatic peroxidation mechanisms in thioacetamide-induced tumors in rats. Effect of a rhodium complex. Carcinogenesis.

[31] Mehendale HM, Roth RA, Gandolfo AJ, Klauning JE, Lemasters JJ, Curtis RL (1994). Novel mechanisms in chemical-induced hepatotoxicity. FASEB J.

[32] Saeter G, Schwarze PE, Nesland JM, Juul N, Pettersen EO, Seglen PO (1988). The polyploidizing growth pattern of normal rat liver is replaced by divisional, diploid growth in hepatocellular nodules and carcinomas. Carcinogenesis.

[33] Sandgren EP, Palmiter RD, Heckel JL, Daugherty CC, Brinster RL, Degen JL (1991). Complete hepatic regeneration after somatic deletion of an albumin-plasminogen activator transgene. Cell.

[34] Fausto N (2004). Liver regeneration and repair: Hepatocytes, progenitor cells, and stem cells. Hepatology.

[35] Michalopoulos GK, DeFrances M (2005). Liver regeneration. Adv. Biochem. Eng. Biotechnol.

[36] Jungermann K, Katz N (1982). Functional hepatocellular heterogeneity. Hepatology.

[37] Baker SJ, Reddy EP (1996). Transducers of life and death: TNF receptor superfamily and associated proteins. Oncogene.

[38] Akerman PA, Cote PM, Yang SQ, McClain C, Nelson S, Bagby G, Diehl AM

[39] (1993). Long-term ethanol consumption alters the hepatic response to the regenerative effects of tumor necrosis factor-alpha. Hepatology.

[40] Xing Z, Richards CD, Braciak T, Thibault V, Gauldie J, Gerok W, Decker K, Andus T, Gross V (1995). Citokine regulation of hepatic acute phase protein espression. Cytokines and the Liver.

[41] Lee CM, Yeoh GC, Olynyk JK (2004). Differential effects of gadolinium chloride on Kupffer cells *in vivo* and *in vitro*. Int. J. Biochem. Cell. Biol.

[42] Bautista AP, Deaciuc IV, Jaeschke H, Schlag G, Redl H (1993). Hepatic responses to bacterial endotoxin (LPS). Pathophysiology of Shock, Sepsis and Organ Failure.

[43] Theocharis SE, Kanelli H, Margeli AP, Spiliopoulou CA, Koutselinis AS (2000). Metallothionein and heat shock protein expression during acute liver injury and regeneration in rats. Clin. Chem. Lab. Med.

[44] Oliver JR, Mara TW, Cherian MG (2005). Impaired hepatic regeneration in metallothionein-I/II knockout mice after partial hepatectomy. Exp. Biol. Med.

